# Where Are We Heading With Fluid Responsiveness and Septic Shock?

**DOI:** 10.7759/cureus.23795

**Published:** 2022-04-03

**Authors:** Mohammed Megri, Emily Fridenmaker, Margaret Disselkamp

**Affiliations:** 1 Pulmonary and Critical Care Medicine, Marshall University Joan C. Edwards School of Medicine, Huntington, USA; 2 Pulmonary and Critical Care Medicine, University of Kentucky College of Medicine, Lexington, USA; 3 Pulmonary and Critical Care Medicine, Veterans Affairs Medical Center, Lexington, USA

**Keywords:** pulse pressure variation, pocus, liberal vs restricted approach to fluid resuscitation in septic shock, passive leg raising, fluid responsiveness

## Abstract

When hypovolemia is left uncorrected, it can lead to poor tissue oxygenation and organ dysfunction. On the other hand, excessive fluid administration can increase the risk of complications. Assessing volume responsiveness in critically ill patients is therefore crucial. In this article we summarized the literature addressing the most sensitive and specific dynamic predictors for fluid responsiveness, to help clarify the best way to guide clinicians in managing patients with shock. Data were collected from PubMed and EMBASE of high-quality articles, randomized controlled trials (RCTs), retrospective research, and metanalyses; articles were identified from January 2000 to February 2021. We identified and critically reviewed the published peer-reviewed literature investigating the dynamic predictors to assess fluid responsiveness. Evidence suggests that the traditional use of static predictors for fluid responsiveness should be abandoned. Over the last 20 years, a number of dynamic tests have been developed. These tests are based on the principle of inducing short-term changes in cardiac preload using heart-lung interactions. However, in routine practice the conditions to meet the requirements of these dynamic parameters are frequently not met. Therefore, more dynamic predictors that do not depend on heart-lung interaction have developed such as the mini fluid challenge test and passive leg raising test These tests have fewer limitations and higher sensitivity and specificity compared to the other tests.

## Introduction and background

The primary goal of fluid administration is to increase the cardiac output (CO) and ultimately the delivery of oxygen to tissues. Patients with acute circulatory failure often do not respond to fluid administration. In fact, nearly half of all critically ill patients in the ICU are not fluid responsive [1]. Nonetheless, hypovolemia can lead to poor tissue oxygenation, resulting in organ dysfunction and death. However, excessive fluid administration is associated with increased length of hospitalization, higher complications, and increased mortality [2,3]. This delicate balance makes determining which patients will be fluid responsive a difficult but important task for critical care clinicians. In this article we will review the most sensitive and specific dynamic predictors of fluid responsiveness, beginning with a review of heart-lung interactions, as understanding this physiology is integral to more reliably predicting a patient’s response to fluid administration or to mechanical ventilation.

## Review

Heart-lung interactions in mechanically ventilated patients: the right heart

Mechanical ventilation exerts a positive intrathoracic pressure (ITP) that varies with inspiration and expiration. Because the heart and lungs occupy the same anatomical location in the thoracic cavity, the heart is essentially a pressure chamber within a pressure chamber. This means that changes in lung volumes and pleural pressures during respiration will affect the dynamics of the cardiac cycle [4]. Flow through a circuit is determined by driving pressures (pressure gradients) within that circuit-changes in pressure gradients between the cardiovascular and pulmonary system during a mechanically ventilated breath will cause variations in stroke volume (SV), pulse pressure (PP) and CO [5-7].

**Figure 1:**
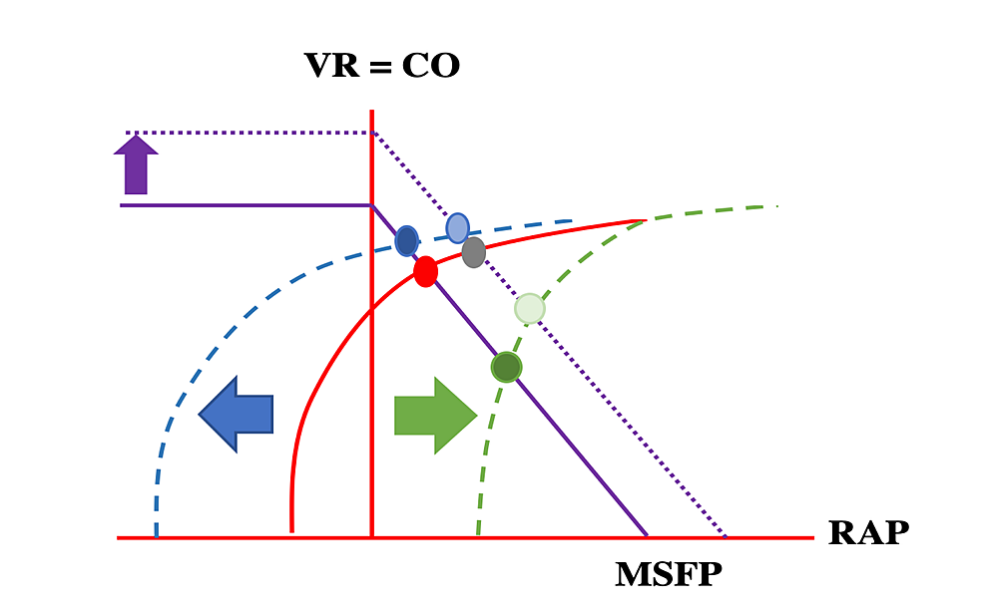
Cardiac function curves: Graphical analysis of the venous return function and cardiac output, with superimposition of the venous return function curve (purple) and the Frank-Starling (cardiac output) curve (red). The intersection between these two curves in an equilibrated system is RAP. Venous return reaches its maximum when the RAP is near zero. The venous return function curve intersects the x axis at zero blood flow, where it represents the MSFP. In the case of volume responsiveness, the VR function curve is shifted upwards and to the right and reaches a new equilibrium (dotted purple line with resulting grey RAP). With spontaneous inspiration, pleural pressure and RAP drop (blue arrow), while transmural RAP will rise. The Starling curve is shifted to the left (dotted blue curve). A new equilibrium point is reached (dark blue RAP), cardiac output will rise despite lower RAP. With mechanical inspiration and positive intrathoracic pressure, the opposite happens (dotted green line). Lower cardiac output despite a higher RAP (dark green) is observed. VR = venous return, CO = cardiac output, MSFP = mean systemic filling pressure, RAP = right atrial pressure Adapted from reference [5].

Preload to the right atrium (RA) and right ventricle (RV) depends on the pressure gradient between the venous “reservoir” and the backflow pressure (right atrial pressure [RAP]). The RAP depends on the ITP, transmural pressure (intramural pressure - extramural pressure), venous elasticity and compliance. The venous reservoir is the volume of blood in the venous circulation, which is predominately located in veins and venules. The blood stretches the vessel walls and depending on the volume and the vessel elastance creates a resting pressure, the mean systemic filling pressure (MSFP) [5-7]. The MSFP is the forward pressure driving the venous return back to the heart. Further lowering of the RAP (as in spontaneous inspiration) increases the pressure gradient for venous return (Figure 1).

This effect is not limitless however, for two reasons: first, veins have floppy walls that will collapse when the pressure inside is less than the pressure outside of the vessel. Second, the right ventricle is limited by capacity. Further increases in venous return do not increase the end-diastolic volume but will increase the end-diastolic pressure [4,7,8].

Venus Return = (MSFP-RAP)/Resistance

In passively ventilated patients, inspiration increases the ITP which will increase the RA pressure but still lower than the pleural pressure, therefore, the RA transmural pressure (RA pressure - pleural pressure) decreases, causing a subsequent increase in RAP; this drops the pressure gradient between the mean systemic filling pressure (superior vena cava [SVC] and inferior vena cava [IVC]) and RA, resulting in a decrease in venous return and distension of the IVC and collapse in the SVC. At the same time, the positive pressure in the alveoli during inspiration increases the pulmonary capillary pressure, which leads to an increase in pulmonary vascular pressure thus increasing the right ventricular afterload [9,10]. To summarize, during inspiration the RV preload decreases, the IVC distends and SVC collapses, and RV afterload increases. This causes a decrease in left ventricle (LV) preload in the following cycle.

Expiration in passively ventilated patients reduces the ITP and RAP compared to inspiration. This increases the transmural pressure in the RA. This increases the pressure gradient between the mean systemic filling pressure and the reservoir, which increases the venous return to the right side of the heart. Despite continued positive end expiratory pressure (PEEP), the pressures inside the alveoli are lower than during inspiration, thus reducing RV afterload, this increases LV preload in the following cycle [4,9].

Heart-lung interaction in mechanically ventilated patients: the left heart

Inspiration increases ITP, which forces blood out of the pulmonary vascular bed and increases LV preload. An increase in ITP also decreases the transmural pressure of the LV. These combined effects decrease the LV afterload, increasing SV and systolic arterial pressures [4,7,8].

The opposite occurs during expiration. LV preload decreases, LV afterload increases compared to inspiration, both of which subsequently decrease the SV [4,7,8].

**Figure 2:**
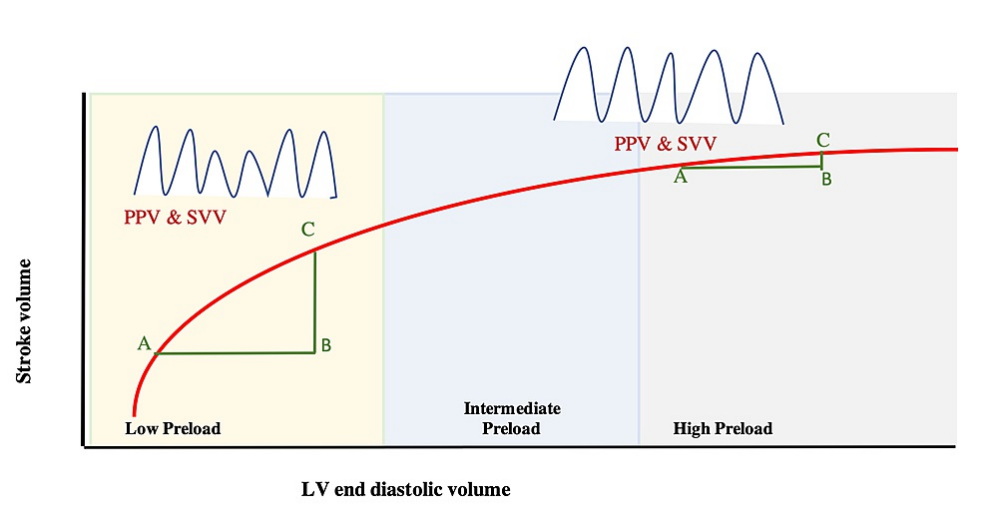
Frank-Starling curve showing the relation between LV stroke volume and left LV end-diastolic volume. At the low preload zone, giving fluid from point A-B increased the SV from point A-C. Giving a fluid bolus in the high preload zone (A-B) made minimal changes in SV (A-C) PPV: Pulse Pressure Variation, SVV: Stroke Volume Variation, LV: left ventricle, SV: stroke volume

The amount of variation in venous return, SV, CO, and PP during mechanical ventilation depends on total lung volume, cardiac rhythm, lung compliance, breathing pattern, and respiratory rate [11]. The changes in intrathoracic pressure and subsequent drop or rise in venous return and SV in part depend on the patient’s location on the Frank-Starling curve. If the patient is on the steep part of the curve (low preload zone) then they will have a significant response to fluid administration (Figure 2).

The opposite is true if the patient is on the far end of the curve; patients located here will be less fluid responsive or not fluid responsive at all (Figure 2). How then can clinicians determine where an individual patient falls on this curve?

Fluid responsiveness

A positive fluid response is generally defined as an increase in cardiac output by >15% after a 500 mL fluid challenge over 10-15 minutes [1]. Testing fluid responsiveness can help clinicians determine when to continue and when to stop fluid administration. It may also help guide physicians when managing fluid removal as well. Fluid responsiveness should be evaluated in patients with acute circulatory shock who have received fluids initially, remain hypotensive (mean arterial pressure <65 mmHg), and have signs of tissue hypoxia.

Dynamic predictors based on heart-lung interaction during respiration

Stroke Volume Variation (SVV) and Arterial Pulse Pressure Variation (PPV) 

**Figure 3:**
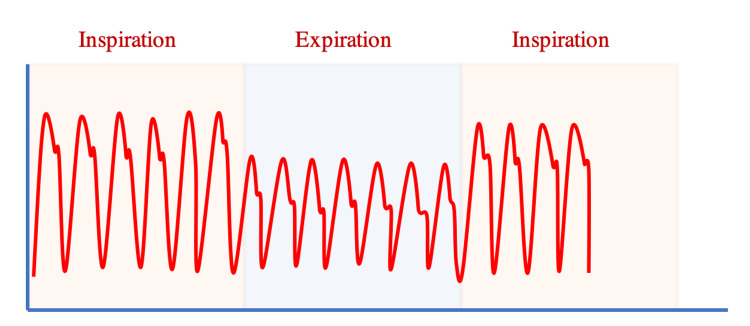
Pulse pressure (PP) variation in an intubated patient during a respiratory cycle. [(Max PP -Minimum PP)/PPmean]x100

SVV and PPV are dynamic physiological parameters. As we have just described, inspiration increases the SV and PP, while expiration decreases these parameters in mechanically ventilated patients. Wide variations in SV and PP may indicate fluid responsiveness. SVV is defined as the difference in maximal SV and minimal SV during respiration. Studies have shown that an SVV threshold >10% indicates fluid responsiveness with 82% sensitivity and 86% specificity [10-15]. Because aortic pulse pressure (aortic systolic pressure - aortic diastolic pressure) directly correlates to left ventricular stroke volume (as a function of central aortic compliance), arterial pulse pressure variation is frequently used as a surrogate for left ventricular SVV [1]. Arterial pulse pressure variation is defined as the difference between the maximum pulse pressure minus the minimum pulse pressure divided by the mean PP during respiration [PPV = 100 x (PPmax - PPmin)/PPmean] (Figure 3).

A PPV threshold of ≥ 13% predicts positive fluid responsiveness (85-89% sensitivity and 88-98% specificity) [10-18]. 

The SVV is the difference between the maximal and minimal SV during respiration. The greater the difference, the more fluid responsive a patient is likely to be [SVV = 100 x (SVmax - SVmin)/SVmean].

Continuous measurement of PPV and SVV can be done invasively, using pulse contour analysis systems. The LiDCO (London, UK), FloTrac (Edwards Lifesciences, Irvine, CA, USA), and Vigileo (Edwards Lifesciences) systems require arterial line placement while the PiCCO system (Getinge, Gothenburg, Sweden) requires a deep arterial line (femoral line, brachial line, or axillary line) [16,17]. Pulse contour analysis is based on the relationships between blood pressure, SV, arterial compliance, and systemic vascular resistance (SVR). All available pulse contour analysis systems use different pressure volume conversion algorithms and are not 100% accurate [10-18]. 

**Figure 4:**
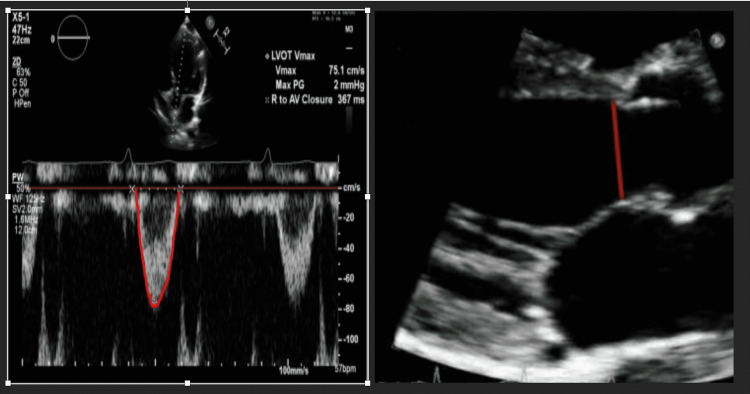
LEFT: Apical five chamber view with illustration of how to measure the LVOT VTI. RIGHT: Parasternal long axis view measuring the LVOT diameter. By using the LVOT VTI and LVOT diameter, stroke volume and cardiac output can be measured [SV = VTI x CSA]. [CSA = 0.785 x (LVOT diameter)]. LVOT: left ventricular out flow, VTI: velocity time interval, SV: stroke volume, CSA: Cross-sectional area

PPV and SVV can also be measured non-invasively, using the Nexfin system (BMEYE, Amsterdam, the Netherlands) or point of care ultrasound (POCUS). To measure SVV with POCUS [15], SV is calculated by measuring the velocity time interval (VTI) using the pulse wave doppler (PWD) and measuring the left ventricular out flow (LVOT) diameter at the same point (Figure 4). 

An SVV of 14% on echocardiogram has a very high positive predictive value for fluid responsiveness, and less than 10% has a high negative predictive value [17]. POCUS carries the added benefit of providing information regarding the etiology of shock. It does have limitations though, such as variation with the operator’s level of skill and with patient body habitus.

Limitations of SVV and PPV

These dynamic indices have poor predictability for fluid responsiveness in patients with low tidal volumes (<8 ml/kg) or low driving airway pressures (<20 cm H_2_O), patients who are spontaneously breathing or triggering the ventilator, patients with cardiac arrhythmias, increased intra-abdominal pressure, right-side heart failure, and patients with high respiratory rates (>35 bpm), and low lung compliance (<30 l/cm) (Table 1).

**Table 1:** Most common physiological limitations to the use of PPV and SVV can be summarized as ‘R-LIMITS’. PPV: Pulse Pressure Variation, SVV: Stroke Volume Variation. Adapted from reference [14].

Heart-Lung Interaction Limitations		
R	Right sided heart failure	False positive
L	Low heart rate (extreme bradycardia) Low respiratory rate (or high rate)	False negative
I	Irregular cardiac rhythm	False positive
M	Mechanical ventilation with low tidal volumes	False negative
I	Increased abdominal pressure	False positive
T	Thorax open	False negative
S	Spontaneous breathing	False positive

Vena cava diameter variation

**Figure 5:**
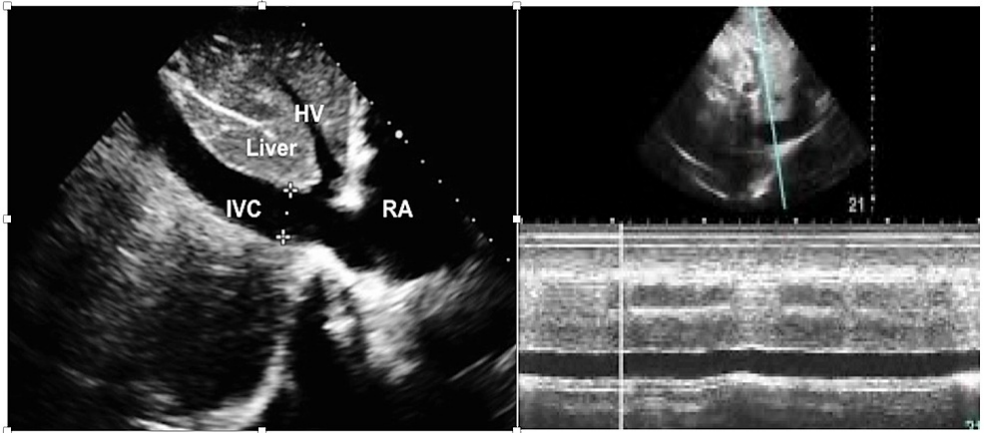
LEFT: Subcostal view of the IVC, hepatic vein, a portion of the liver, and the RA with IVC diameter measured. RIGHT: Using M-Mode to measure the distensibility of the IVC. IVC: inferior vena cava, RA: right atrium, HV: hepatic vein

Because there is no valve between the vena cava and right atrium, distension of the vena cava is thought to correlate with increased right atrial pressure. In a spontaneously breathing patient, the IVC collapses during inspiration. Conversely, in a patient supported with positive pressure ventilation, the IVC distends during inspiration. The magnitude of the change in diameter has been proposed to correlate with intravascular volume status and fluid responsiveness. Using POCUS, the IVC was examined subcostally in longitudinal section. Its diameter was measured in M-mode coupled to two-dimensional mode, just above the origin of the suprahepatic vein (approximately 2 cm from the junction of the RA and IVC). Measurements were validated when the M-mode tracing was exactly perpendicular to the IVC (Figure 5) [19-21].

The distensibility index of the IVC (dIVC), which reflects the increase in its diameter on inspiration, was calculated as (maximum diameter on inspiration - minimum diameter on expiration) / minimum diameter on expiration. A distensibility index above 18% favors fluid responsiveness (63% sensitivity and 73% specificity) [19-21].

**Figure 6:**
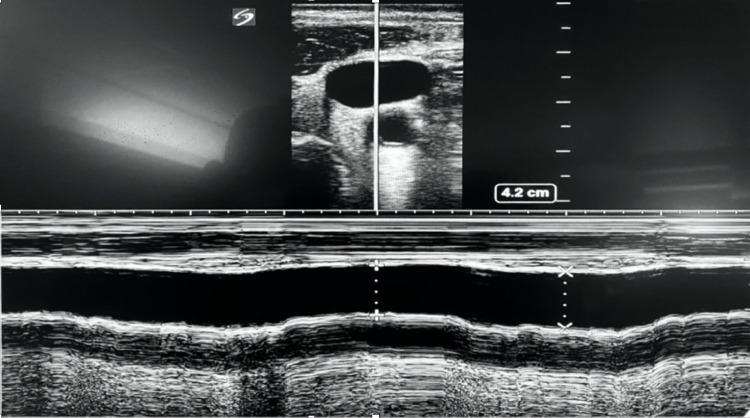
Jugular vein variation in intubated patient showing distensibility index of 13%, which suggests that the patient is not fluid responsive

Measuring the distensibility of the internal jugular vein (dIJV) has also been used to determine fluid responsiveness in critically ill patients (Figure 6). IJV distensibility assesses venous return and right ventricular reserve. Distensibility of IJV of 18% has a sensitivity of 80% and a specificity of 85% in mechanically ventilated patients [22]. However, the dIVC and dIJV still carry the same limitations: they are inaccurate with low tidal volumes (<8 ml/kg) or low driving airway pressures (<20 cm H_2_O), spontaneous breathing, increased intra-abdominal pressure (for dIVC), right-sided heart failure, high respiratory rates (>35 bpm), and low lung compliance (<30 l/cm) (Table 1). Their advantages are that compared to SVV and PPV, the dIVC and dIJV are not affected by cardiac arrhythmias and do not require invasive procedures. 

Tidal volume challenge

Most of the dynamic variables such as PPV and SVV were tested using 8-10 ml/Kg tidal volume. After recent landmark studies though, most critically ill patients on medical services are ventilated using a lung protective, low tidal volume (6-8 ml/kg) approach [23]. In order to overcome this limitation, Myatra et al. tested the tidal volume challenge maneuver. To perform this maneuver, the tidal volume is increased from 6 ml/kg IBW to 8 ml/kg for one minute. Changes in PPV and SVV are then evaluated. An absolute change in PPV of 3.5% or SVV of 2.5% after performing this maneuver predicts fluid responsiveness with 75-88% sensitivity and 93-100% specificity [24-26].

Limitations are that this can be inaccurate with low driving airway pressures (<20 cmH_2_O), spontaneous breathing, cardiac arrhythmias, increased intra-abdominal pressure, right-sided heart failure, high respiratory rates (>35 bpm), and low lung compliance (<30 l/cm) (Table 1).

End-expiratory occlusion test

Performing a 15-second expiratory hold and preventing cycling and inspiration will result in a decrease in venous return and cardiac preload. During an expiratory hold, the pressure gradient between the SVC, IVC, and RA will increase. In fluid responsive patients, this can increase the CO by 5% [27-29]. Because this difference is slight, this test requires precise, accurate, and continuous measurement of CO (such as pulse contour analysis using an arterial line and a LiDCO, Flow trac, or Vigileo system). Changes in CO >5% using pulse contour analysis and the end-expiratory hold test can detect fluid responsiveness with a sensitivity and specificity of 90%. Adding echocardiogram evaluation of CO to this test has demonstrated that changes of LVOT-VTI (Figure 5) of ≥9% can reliably detect fluid responsiveness with sensitivity of 89% and specificity of 95% (though standard POCUS limitations like operator skill and patient body habitus can impact the usefulness of this addition) [27-29].

Limitations are inaccuracy in patients with tidal volume <8 ml/kg or PEEP >10 cmH_2_O. Advantages are that it can be used in patients with cardiac arrhythmias and patients who are triggering the ventilator. 

Dynamic predictors not based on heart-lung interaction

Mini-Fluid Challenge Test

To perform a mini-fluid challenge, a small volume of crystalloid or colloid is infused intravenously over a predetermined amount of time, and the changes in the patient’s LVOT-VTI, CO, PPV, and SVV are assessed. Initially the mini-fluid challenge was performed in mechanically ventilated, critically ill patients in acute circulatory failure with 100 mL of fluid over one minute. A change of >10% in LVOT-VTI on five-chamber view using echocardiogram (Figure 5) reliably detected fluid responsiveness with sensitivity of 95% and specificity of 78% [30,31]. Another study was conducted administering 50 ml of crystalloid or colloid fluid over 10 seconds. A change in CO of >6% had a 93% sensitivity and 91% specificity, and change in LVOT-VTI of 9% had a sensitivity of 74% and specificity of 95% for detecting fluid responsiveness [30,31]. In clinical practice however, major limitations of this technique include ultrasound availability, physician skill in echocardiography, and poor echogenicity particularly in mechanically ventilated patients. In order to overcome these limitations, a study using pulse contour analysis was performed using a 100 mL fluid bolus and assessment of PPV and SVV. Changes in 4% and 3% respectively had sensitivities of 86% and specificities of 89% [30]. Limitations include cardiac arrhythmia.

Passive Leg Raising Test (PLRT)

**Figure 7:**
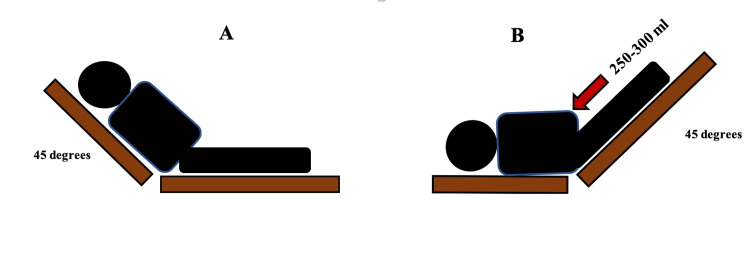
A: Initial position before the passive leg raising (PLR) test. B: Position of the patient at the end of PLR

Passive leg raising (PLR) creates a reversible increase in venous return allowing for prediction of fluid responsiveness without committing the patient to a fluid bolus. Similar to the mini-fluid challenge test, the PLR must be combined with invasive or non-invasive measures of cardiac output. PLR is essentially an administration of 150-300 mL of fluid by transferring this volume from the lower part of the body to the right side of the heart [32]. This hemodynamic effect is rapidly reversible by returning the patient back to the original position [32,33]. PLR should start from a recumbent position (Figure 7A). The patient is then moved to a supine position with the legs elevated to 45 degrees (Figure 7B). CO and SV should be measured before, during, and after the PLR test [32-34].

The measurement of CO and SV with PLRT can be done invasively using the pulse contour analysis or non-invasively using echocardiogram, end-tidal carbon dioxide (ETCO2), or bioreactance (NICOM device) [34-38]. SVV on echocardiogram can be established by measuring the LVOT diameter and the VTI (Figure 4) but must be obtained before and during the test. Major limitations of this technique include ultrasound availability and physician skill in echocardiography. Thoracic bioreactance is a non-invasive way to measure cardiac output. It consists of four electrodes connected to a monitor with two electrodes on each side of the body. The electrodes on a given side of the body are paired, so the currents are passed between the outer electrodes of the pair and voltages are recorded from between the inner electrodes. Thus, a noninvasive CO measurement signal is determined separately from each side of the body, and the final noninvasive CO measurement signal is obtained by averaging these two signals. The bioreactance CO measurement is highly correlated with the CO measured by thermodilution and pulse contour analysis [39,40]. A threshold of 10% change in CO or SV reliably predicts fluid responsiveness with sensitivity of 85% and specificity of 91% [41,42]. The advantage of bioreactance is that it can calculate the CO, CI (cardiac index), SV and provide the patient’s location on the Frank-Starling curve. A recent prospective, multicenter, randomized clinical trial at 13 hospitals evaluated fluid resuscitation guided by PLR which revealed that using PLR to evaluate fluid responsiveness in septic shock patients reduced the net fluid balance and reduction in the risk of renal and respiratory failure, and may improve outcome in patient with septic shock compared to usual care [42]. ETCO2 variation with PLR test can also predict fluid responsiveness. A 5% increase in ETCO2 after PLR predicts fluid responsiveness with sensitivity of 71% and specificity of 95-100% [35]. Some simple precautions must be taken to avoid false results: the PLR test should be performed by adjusting the bed and not the patient’s legs, airway secretions should be suctioned prior to performing the test, and if the patient is awake the test should be explained to the patient beforehand. Of note, if using PPV with the PLR, PPV >12% at the end of the test in spontaneously breathing or mechanically ventilated patients predicted fluid responsiveness with sensitivity of 58% and specificity of 83%. Changes in pulse pressure on PLR exhibited a lower diagnostic performance compared with PLR-induced changes in CO and SV. PLR-guided resuscitation resulted in a significantly lower net fluid balance and reduced renal and respiratory dysfunction in sepsis and septic shock patients [36-38].

Its limitations are that it cannot be used in patients with head trauma, and some studies suggest that high intra-abdominal pressure can lead to false-negative results [43]. Advantages are that the test is accurate with low tidal volume (<8 ml/Kg), low driving airway pressures (<20 cm H_2_O), spontaneous breathing, patient triggered ventilation, cardiac arrhythmias, high respiratory rates (>35 bpm), and low lung compliance (<30 l/cm). 

**Figure 8:**
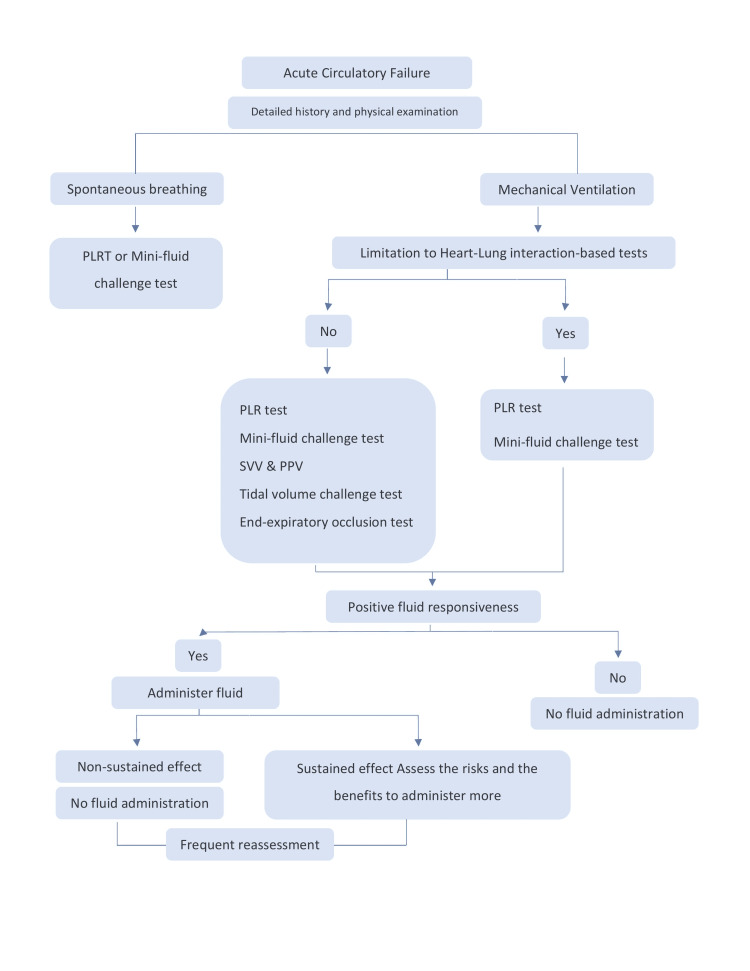
Approach to predict fluid responsiveness in critically ill patients PPV: Pulse pressure variation, SVV: stroke volume variation, PLR: Passive leg raising

As careful use of all the dynamic predictors of fluid responsiveness can help clinicians find the delicate balance between benefit and harm, in Figure 8 we summarize the fluid responsiveness step by step.

## Conclusions

Fluid administration is a cornerstone in the treatment of critically ill patients with clinical signs of hypoperfusion, but fluid overload is associated with increased mortality. Dynamic predictors and tests can be added to a detailed history and a close examination in order to guide fluid resuscitation. A strong understanding of the patient’s individual physiology is paramount to using these methods successfully, as most dynamic predictors and tests are based on heart-lung interactions (particularly in the context of mechanical ventilation). As with all diagnostic tests, the limitations of each must be considered in order to avoid incorrect interpretations of the results. Furthermore, positive fluid responsiveness does not mean one must administer fluid; at every decision point one must weigh the risks and the benefits of fluid administration based on the individual patient’s unique physiology and circumstances. Careful use of these dynamic predictors of fluid responsiveness can help clinicians find the delicate balance between benefit and harm.
